# Nevirapine, Sodium Concentration and HIV-1 RNA in Breast Milk and Plasma among HIV-Infected Women Receiving Short-Course Antiretroviral Prophylaxis

**DOI:** 10.1371/journal.pone.0121111

**Published:** 2015-03-26

**Authors:** Kirsten Salado-Rasmussen, Zahra P. Theilgaard, Mercy G. Chiduo, Ib C. Bygbjerg, Jan Gerstoft, Margrethe Lüneborg-Nielsen, Martha Lemnge, Terese L. Katzenstein

**Affiliations:** 1 Department of Infectious Diseases, Copenhagen University Hospital, Rigshospitalet, Copenhagen, Denmark; 2 National Institute for Medical Research, Tanga, Tanzania; 3 Department of International Health, Immunology and Microbiology, Faculty of Health Sciences, University of Copenhagen, Copenhagen, Denmark; University of British Columbia, CANADA

## Abstract

**Introduction:**

Risk factors for breast milk transmission of HIV-1 from mother to child include high plasma and breast milk viral load, low maternal CD4 count and breast pathology such as mastitis.

**Objective:**

To determine the impact of nevirapine and subclinical mastitis on HIV-1 RNA in maternal plasma and breast milk after intrapartum single-dose nevirapine combined with either 1-week tail of Combivir (zidovudine/lamivudine) or single-dose Truvada (tenofovir/emtricitabine).

**Methods:**

Maternal plasma and bilateral breast milk samples were collected between April 2008 and April 2011 at 1, 4 and 6 weeks postpartum from HIV-infected Tanzanian women. Moreover, plasma samples were collected at delivery from mother and infant.

**Results:**

HIV-1 RNA was quantified in 1,212 breast milk samples from 273 women. At delivery, 96% of the women and 99% of the infants had detectable nevirapine in plasma with a median (interquartile range, IQR) of 1.5 μg/mL (0.75–2.20 μg/mL) and 1.04 μg/mL (0.39–1.71 μg/mL), respectively (P < 0.001). At 1 week postpartum, 93% and 98% of the women had detectable nevirapine in plasma and breast milk, with a median (IQR) of 0.13 μg/mL (0.13–0.39 μg/mL) and 0.22 μg/mL (0.13–0.34 μg/mL), respectively. Maternal plasma and breast milk HIV-1 RNA correlated at all visits (R = 0.48, R = 0.7, R = 0.59; all P = 0.01). Subclinical mastitis was detected in 67% of the women at some time during 6 weeks, and in 38% of the breast milk samples. Breast milk samples with subclinical mastitis had significantly higher HIV-1 RNA at 1, 4 and 6 weeks (all P < 0.05).

**Conclusion:**

After short-course antiretroviral prophylaxis, nevirapine was detectable in most infant cord blood samples and the concentration in maternal plasma and breast milk was high through week 1 accompanied by suppressed HIV-1 RNA in plasma and breast milk.

## Introduction

Transmission of HIV-1 through breast milk is a concern in many developing countries where the HIV-1 prevalence is high and breastfeeding is common. A study from Kenya, where women were randomly assigned to either breastfeeding of formula feeding, demonstrated that more than one-third of the transmission was attributable to breastfeeding [1]. However, non-breastfed infants in developing countries have an increased risk of diarrheal disease, respiratory illness, malnutrition and increased mortality [2]. In places where replacement feeding is not acceptable, feasible, affordable, sustainable and safe breastfeeding has been recommended by the World Health Organization (WHO) to HIV-infected women [3]. However, in recent WHO guidelines the impact of antiretroviral (ARV) prophylaxis during the breastfeeding period is recognized and recommended [4].

Pathogenesis of HIV-1 transmission through breastfeeding is not completely understood but certain risk factors, including low maternal CD4 count, high plasma or breast milk HIV-1 RNA and mastitis have been associated with increased transmission [5–7]. Mastitis increases the permeability of the mucosal barrier of the mammary epithelium and sodium (Na^+^) passes into the breast milk, resulting in elevated Na^+^ concentrations in the breast milk [8–10]. In absence of clinical symptoms, the term subclinical mastitis is used for this condition, whereas clinical mastitis is characterized by engorgement or darkening of the breast, pain and fever. A recent study directly measured changes in breast milk HIV-1 RNA in women with clinical mastitis or abscesses and found that the breast milk viral load increased modestly in the affected breast and returned to pre-pathology levels after symptom resolution [11]. Further, the study showed that the viral load in the contralateral breast was not affected [11]. Likewise, subclinical mastitis has also been associated with elevated breast milk HIV-1 RNA [12–15]. WHO recommends that HIV-infected women with clinical mastitis who are breastfeeding stop breastfeeding from the affected breast and feed from the uninfected breast only (mastitis is most often unilateral) until symptoms resolve [16].

Recently, a pooled individual data analysis of five randomized trials of infant nevirapine (NVP) prophylaxis to prevent breast-milk HIV-1 transmission indicated that daily treatment of infants for 28 weeks reduced breast-milk HIV-1 transmission by 71% [17]. Moreover, it has been pointed out that the ideal infant-only strategy should contain drugs less likely than NVP to select resistant virus that can compromise the treatment of those infants who become infected despite prophylactic treatment [18].

Our main study was designed to assess the development of NVP resistance after two short-course regimens designed to reduce the risk of NVP resistance. In the light of the latest strategies for prevention of mother-to-child transmission (PMTCT) of HIV, now focusing on treatment of all breastfeeding mothers regardless of CD4 count or extended infant prophylaxis, the single-dose regimens seem to have less relevance. However, documentation of feasibility, acceptability and safety for these extended regimens is still needed. Moreover, some countries only reach <50% of the patients who need antiretroviral therapy (ART) for their own health [19], and stock-outs of ARVs may occur, in which cases NVP may have a role as back-up.

The aim of the present study was to determine the impact of NVP and subclinical mastitis on HIV-1 RNA in maternal plasma and breast milk after intrapartum single-dose NVP combined with either a 1-week tail of Combivir (zidovudine/lamivudine) or single-dose Truvada (tenofovir/emtricitabine).

## Methods

### Study Population and Sample Collection

Plasma and breast milk samples were collected from HIV-infected women enrolled in a randomized clinical trial which aimed to evaluate mother-to-child transmission (MTCT) of HIV and nonnucleoside reverse transcriptase inhibitor (NNRTI) resistance development among women receiving single-dose NVP plus an additional drug (Backup with Combivir or single-dose Truvada in order to avoid NNRTI resistance after single-dose nevirapine for the prevention of mother-to-child transmission (The ComTru Study); ClinicalTrials.gov number NCT00346567). In brief, pregnant women attending Reproductive and Child Health clinics at Bombo Regional Hospital, Ngamiani Health Centre and Makorora Health Centre situated in Tanga, Tanzania were voluntarily tested for HIV-1. The included women were HIV-positive, aged 18 or above and were antiretroviral naïve. They agreed to deliver at the hospital or health centre and to stay in the catchment area within the follow-up period. Women fulfilling the national criteria for ART were excluded and referred to the Care and Treatment Clinic at Bombo Regional Hospital, Ngamiani Health Centre or Makorora Health Centre for further monitoring and treatment. Women suffering from systemic diseases with blood creatinine >1.5 mg/dL or with blood alanine aminotransferase concentration >140 U/L were excluded. Women were randomized to single-dose NVP plus an additional study drug (i.e. Combivir twice daily for seven days or single-dose Truvada). The neonates were given a single oral dose of 2 mg/kg NVP suspension within 72 hours of birth. Women enrolled after September 2008 also received antepartum zidovudine (AZT) from week 28 gestational age and their infants received AZT twice daily for 7 or 28 days in accordance with the revised WHO guidelines [20]. This report is confined to the analysis of the breast milk samples; the rate of MTCT and NNRTI resistance development will be reported separately (Z. Theilgaard et al, in preparation). For the current study we included all women from the ComTru study who provided at least one breast milk sample.

Plasma and bilateral breast milk samples were collected between April 2008 and April 2011 at 1, 4 and 6 weeks postpartum. Moreover, plasma samples were collected at delivery from mother and infant (cord blood). The women were asked to provide 15 mL of breast milk from each breast. Study nurses registered any breast problems i.e. cracked nipples, paucity of milk, abscess or pain, and categorized it to the right or left side, respectively. Information on breast feeding practice was collected and categorized, i.e. exclusive breastfeeding or mixed feeding.

### Measurement of Plasma and Breast Milk NVP Concentration

We measured NVP concentration in all available plasma samples collected from the infants and mothers at time of delivery, and in all available maternal breast milk and plasma samples collected at 1 week postpartum. Furthermore, we measured NVP concentration in a subset of maternal plasma samples from 4 weeks postpartum. Samples beyond 4 weeks postpartum were not analyzed, because at this time the NVP concentration after single-dose NVP is extremely low [21]. Plasma and breast milk NVP concentrations were determined by the ARK NVP-test. The ARK NVP-test is a rapid automated enzyme immunoassay based on competitive binding to antibody between drug from the sample and drug-labelled enzyme. The method requires no sample pre-treatment and has shown good correlation with high-performance liquid chromatography (HPLC) [22] and has been validated for use on human breast milk [23]. The ARK NVP-test was performed using COBAS MIRA Plus (Roche Molecular Systems Inc., Branchburg, NJ, USA) using 16 μL plasma or breast milk for duplicate measurements.

### Measurement of Plasma and Breast Milk HIV-1 RNA

We measured HIV-1 RNA on all available plasma samples using the Roche AMPLICOR Monitor test kit version 1.5 (Roche Molecular Systems Inc., Branchburg, NJ, USA) or the Roche COBAS AmpliPrep/Cobas TaqMan 48 test kit version 2.0 (Roche Molecular Systems Inc., Branchburg, NJ, USA), both methods having a lower limit of detection of 20 copies/mL. We determined HIV-1 RNA in all available breast milk samples following the procedure described by Salazar-Gonzalez et al [24] with some modifications. In brief, breast milk samples were diluted 1:5 in phosphate-buffered saline (PBS) and centrifuged at 17,000 g for 1 hour at 4°C. The upper lipid layer was then carefully removed and discarded. The cell pellet was resuspended in the supernatant. We used the Roche COBAS AmpliPrep/Cobas TaqMan 48 test kit version 2.0 (Roche Molecular Systems Inc., Branchburg, NJ, USA). The lower limit of detection was 100 copies/mL due to the dilution step.

### Measurements of Breast Milk Na^+^ Concentration

As a laboratory indicator of mastitis, Na^+^ concentration in breast milk supernatant was determined in all available breast milk samples using a Modular Analytics SWA (Roche Diagnostics GmbH, Mannheim, Germany), with a lower limit of detection of 10 mmol/L. In brief, 1 mL of the whole breast milk sample was centrifuged at 1,300 g for 7 minutes, and the lipid and aqueous portions were separated. The lipid fraction was removed and the cell pellet was resuspended in the aqueous portion. The aqueous portion was analyzed in duplicates by the use of ion-selective electrodes [5]. Breast milk Na^+^ concentration was defined as elevated at concentrations >12 mmol/L (>3SD above the mean for normal human milk) [5;25].

### Statistical Analysis

Log_10_ transformation was done on the HIV-1 RNA results from plasma and breast milk to obtain an approximate normal distribution. For statistical analysis, plasma HIV-1 RNA less than 20 copies/mL were assigned a value of 10 copies/mL and breast milk HIV-1 RNA less than 100 were assigned a value of 50 copies/mL (the mid-point between zero and the limit of detection of the assay). Nevirapine concentrations below 0.25 μg/mL were assigned a value of 0.125 μg/mL (the mid-point between zero and the limit of detection of the assay). Na^+^ concentration was examined both as a dichotomous variable (i.e., normal/elevated) and as a continuous numeric variable (in mmol/L). Na^+^ concentration values below 10 mmol/L were assigned a value of 5 mmol/L (the mid-point between zero and the limit of detection of the assay). Missing values were excluded from the analyses.

The Wilcoxon signed rank test was used for non-parametric comparisons of NVP concentrations from infants and women, for comparisons of NVP concentrations in plasma and breast milk and for comparisons of Na^+^ when expressed as a continuous variable. The t test was used for comparison of HIV-1 RNA means and the Mann-Whitney test was used to compare plasma and breast milk HIV-1 RNA between groups with and without subclinical mastitis. Spearman’s correlation coefficient was used to describe relations between continuous variables. All p-values were two-tailed. Data analysis was done using SPSS version 16.0 (SPSS Inc., Chicago, Il, USA).

### Ethical Statement

Written informed consent was obtained from all women participating in the ComTru Study. Further, written informed consent was obtained from the mothers on behalf of the participating infants. Ethical approval was given by the Medical Research Coordination Committee of the Tanzanian National Institute for Medical Research and consultative approval was granted by the Development-Country Committee of the Danish National Committee on Biomedical Research Ethics.

## Results

### Study Population

**Fig 1:**
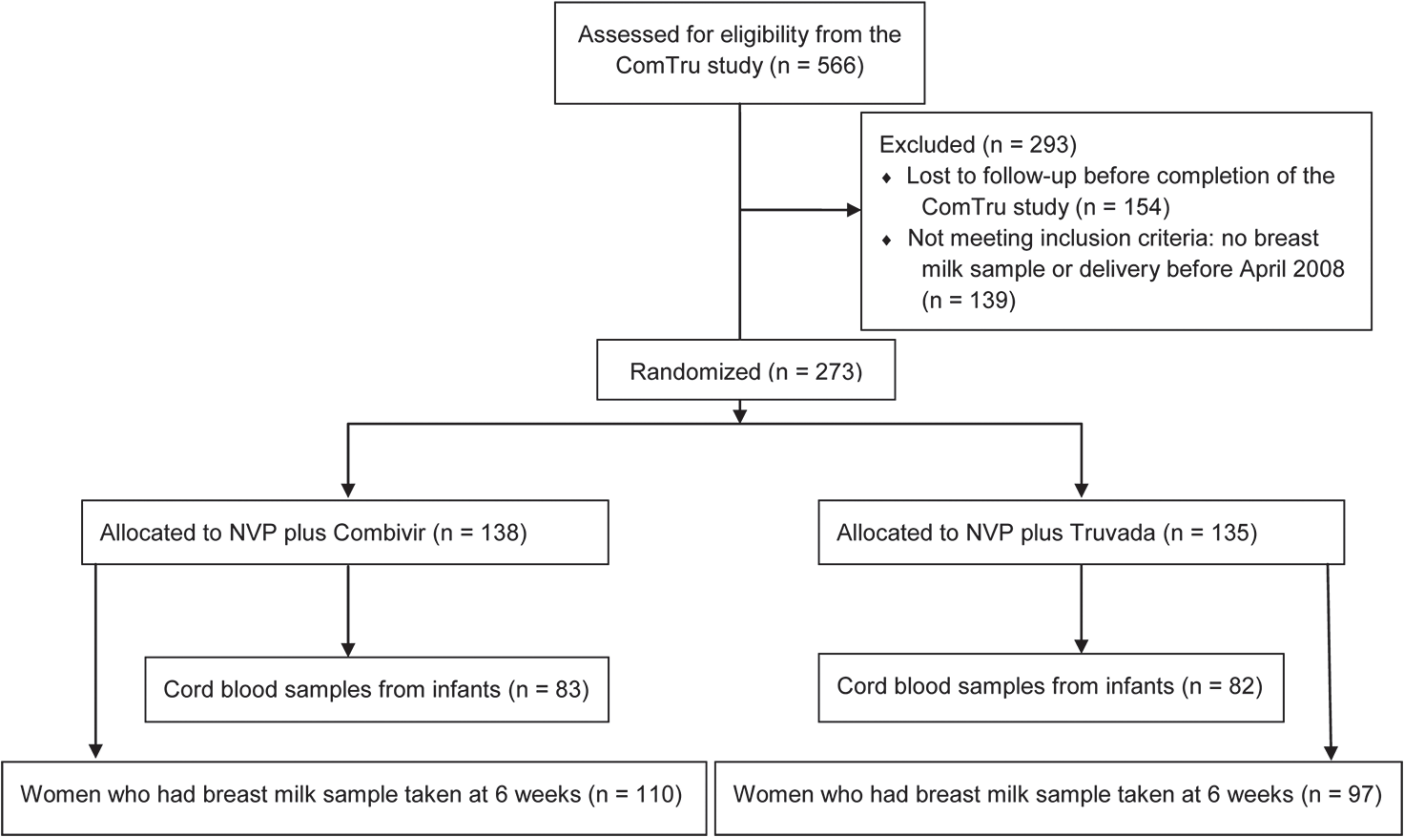
CONSORT Diagram. The flow chart indicates the number of women included from the ComTru study. The women were allocated to Combivir (zidovudine/lamivudine) or Truvada (tenofovir/emtricitabine) treatment before inclusion in the present study. There were no differences in nevirapine concentration, HIV-1 RNA or sodium concentration between the two trial arms of the ComTru study.

A flow diagram of the study is shown in Fig. 1. For the current study we included all women from the ComTru study who provided at least one breast milk sample. A total of 273 women fulfilled this criterion and were included (Table 1). Moreover, we included infants of the above-mentioned women. A total of 1,212 breast milk samples were analyzed for HIV-1 RNA and Na^+^ concentration. Further, all breast milk samples collected at 1 week postpartum were analyzed for NVP concentration.

**Table 1:** Descriptive Characteristics of 273 HIV-Infected Women.

Maternal Age, mean (sd)		27.7 (5.1)
Parity, mean (sd)		1.7 (1.3)
Gravida, mean (sd)		2.8 (1.3)
Education	Primary (%)	234 (86)
	Secondary (%)	29 (11)
	College/university (%)	1 (0)
	None (%)	8 (3)
	No answer (%)	1 (0)
Job	Housewife (%)	225 (82)
	Public servant (%)	11 (4)
	Self-employed (%)	31 (11)
	Jobless (%)	3 (1)
	Other (%)	2 (1)
	No answer (%)	1 (1)
Marital Status	Single (%)	21 (8)
	Married (%)	223 (82)
	Widowed (%)	4 (1)
	Divorced (%)	4 (1)
	Cohabitant (%)	19 (7)
	No answer (%)	2 (1)
Religion	Muslim (%)	55 (20)
	Christian (%)	218 (80)
CD 4 (cells/μL), mean (sd)		435 (170)

### Nevirapine Concentration in Plasma and Breast Milk in Women Receiving Short-Course ARV Prophylaxis

**Fig 2:**
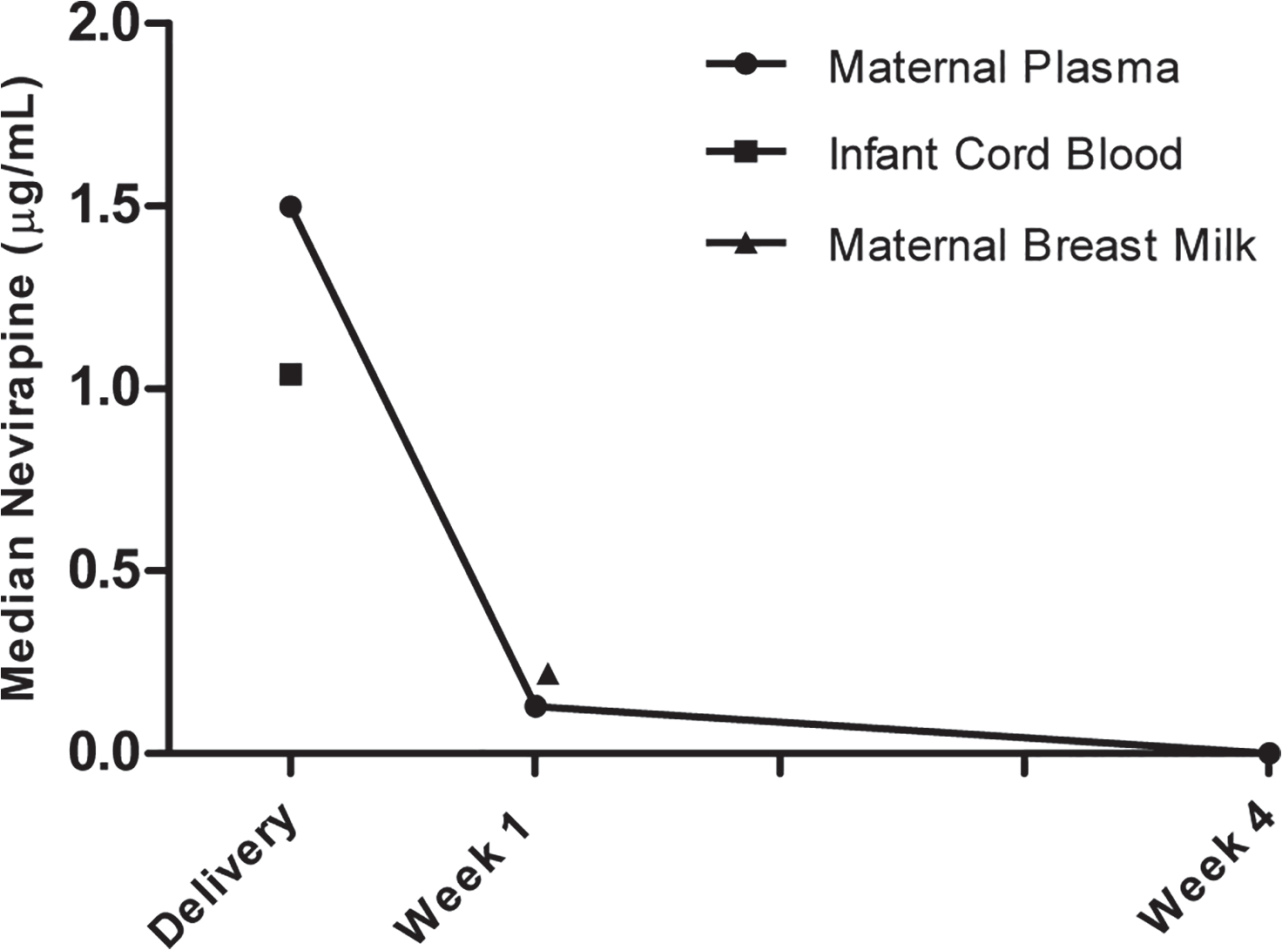
Nevirapine Concentration in Infant Cord Blood and Maternal Plasma and Breast Milk from 273 HIV-Infected Women and their Infants. The medians at delivery are based on 223 maternal plasma samples and 165 infant cord blood samples. The medians at 1 week postpartum are based on 228 maternal plasma samples and 205 breast milk samples. The median at 4 weeks postpartum is based on 42 maternal plasma samples.

Of the 273 women participating in this study, 224 reported taking NVP at time of delivery and two reported not taking NVP at time of delivery. However, data were missing from 47 women. At delivery, 214/223 (96%) of the women with available plasma samples had detectable NVP in plasma with a median (interquartile range, IQR) of 1.5 μg/mL (0.75–2.20 μg/mL) (Fig. 2). Of the nine women with undetectable NVP in plasma at delivery, eight reported taking NVP at onset of delivery and for one woman this data was not collected. Of the infants with available cord blood samples, 164/165 (99%) had detectable NVP with a median (IQR) of 1.04 μg/mL (0.39–1.71 μg/mL) (Fig. 2). The NVP concentration was significantly higher in maternal plasma compared to infant cord blood when analyzing paired mother-infant samples (P < 0.001). At 1 week postpartum, 212/228 (93%) of the women had detectable NVP in plasma with a median (IQR) of 0.13 μg/mL (0.13–0.39 μg/mL) (Fig. 2). Further, 200/205 (98%) of the women had detectable NVP in at least one breast milk sample at 1 week postpartum, when testing both the right and the left breast milk samples, and the median (IQR) was 0.22 μg/mL (0.13–0.34 μg/mL) (Fig. 2). A subset of maternal plasma samples from 4 weeks postpartum was measured and traces of NVP were found in 33/42 (79%) of the samples. Nonetheless, the NVP concentration was below the lower limit of detection in all 42 samples. The rapid decline in NVP concentration is shown in Fig. 2. Nevirapine concentration in plasma and breast milk correlated at 1 week postpartum (R = 0.83, P = 0.01) (the analysis was done with the right and the left breast milk samples, respectively, and with the average, with the same result). Nevirapine concentration in maternal plasma and infant cord blood also correlated (R = 0.71, P = 0.01). The 200 mg NVP oral dose given to the women at delivery resulted in cord blood concentrations above the 0.10 μg/mL target (10 times the *in vitro* IC_50_ against HIV-1) [26] in 133/165 (81%) of the infant samples.

### HIV-1 RNA in Plasma and Breast Milk in Women Receiving Short-Course ARV Prophylaxis

**Fig 3:**
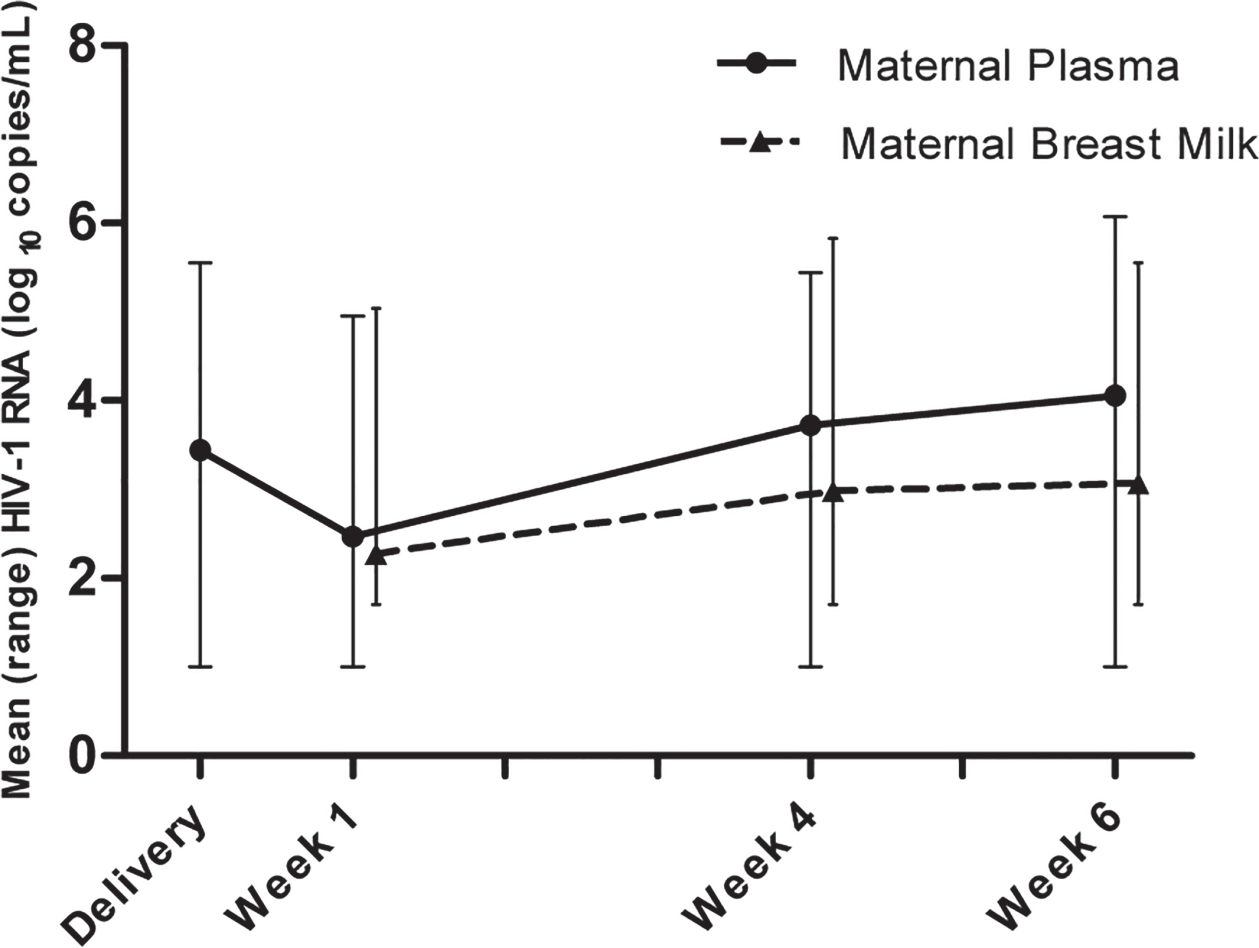
Mean log_10_ HIV-1 RNA in Maternal Plasma and Breast Milk from 273 HIV-Infected Women. Ranges are represented with error bars. The mean at delivery is based on 223 plasma samples. The means at 1 week postpartum are based on 227 plasma samples and 207 breast milk samples. The means at 4 weeks postpartum are based on 228 plasma samples and 198 breast milk samples. The means at 6 weeks postpartum are based on 252 plasma samples and 208 breast milk samples.

Plasma HIV-1 RNA dropped significantly after short-course ARV prophylaxis was given at time of delivery (Table 2). At time of delivery, the mean plasma level of HIV-1 RNA was 3.44 log_10_ copies/mL (range, 1.00 to 5.55 log_10_ copies/mL), and declined at 1 week to a mean level of 2.47 log_10_ copies/mL (range, 1.00 to 4.95 log_10_ copies/mL) (P < 0.001). The breast milk HIV-1 RNA mean level at 1 week postpartum was 2.28 log_10_ copies/mL (range, 1.70 to 5.04 log_10_ copies/mL). After week 1, the viral load rebounded in both plasma and breast milk (Fig. 3). At 1 week postpartum, 51% (106/207) of the women had detectable HIV-1 RNA in breast milk from minimum 1 breast. This number increased to 68% (134/198) at 4 weeks and to 77% (160/208) at 6 weeks postpartum. Maternal plasma and breast milk HIV-1 RNA correlated at 1 week (R = 0.48, P = 0.01), 4 weeks (R = 0.7, P = 0.01) and 6 weeks (R = 0.59, P = 0.01) postpartum. These analyses were done with the right and the left breast milk samples, respectively, and on the average of the right and left breast milk samples with the same result. Breast milk HIV-1 RNA from the right and left breast side, were compared as paired samples and correlated at 1 week, 4 weeks and 6 weeks postpartum (R = 0.45, R = 0.69, R = 0.72; all P = 0.01). However, we found a significant difference in HIV-1 RNA loads between the right and left breast side at 4 and 6 weeks postpartum (P = 0.016 and P = 0.003, respectively). In contrast, we did not find a significant difference in HIV-1 RNA loads from the right and the left breast side, at 1 week postpartum, probably caused by the impact of the ARV prophylaxis on viral load through week 1, resulting in undetectable HIV-1 RNA in about half of the breast milk samples.

**Table 2:** Plasma and Breast Milk HIV-1 RNA through 6 Weeks after Short-Course Antiretroviral Prophylaxis in 273 HIV-Infected Women.

	Plasma	Breast Milk Right	Breast Milk Left	Breast Milk Average
	HIV-1 RNA	HIV-1 RNA	HIV-1 RNA	HIV-1 RNA
	(log_10_ copies/mL)	(log_10_ copies/mL)	(log_10_ copies/mL)	(log_10_ copies/mL)
Delivery	n = 223	N/A	N/A	N/A
Mean, (range)	3.44 (1.00–5.55)			
1 Week)	n = 227	n = 205	n = 202	n = 207
Mean, (range	2.47 (1.00–4.95)	2.21 (1.70–5.22)	2.14 (1.70–5.02)	2.28 (1.70–5.04)
4 Weeks	n = 228	n = 197	n = 194	n = 198
Mean, (range)	3.72 (1.00–5.44)	2.90 (1.70–5.78)	2.71 (1.70–5.90)	2.98 (1.70–5.82)
6 Weeks	n = 252	n = 204	n = 207	n = 208
Mean, (range)	4.06 (1.00–6.07)	3.03 (1.70–5.66)	2.84 (1.70–5.64)	3.07 (1.70–5.55)

N/A Not applicable.

When analyzing paired samples, HIV-1 RNA was significantly higher in plasma compared to breast milk over 6 weeks (all P < 0.001). We found no correlation between HIV-1 RNA and NVP in neither infant cord blood nor maternal plasma or breast milk, except from at 1 week postpartum where HIV-1 and NVP concentrations were inversely correlated in maternal plasma (R = -0.14, P = 0.03).

### Prevalence of Subclinical Mastitis in Women Receiving Short-Course ARV Prophylaxis

**Fig 4:**
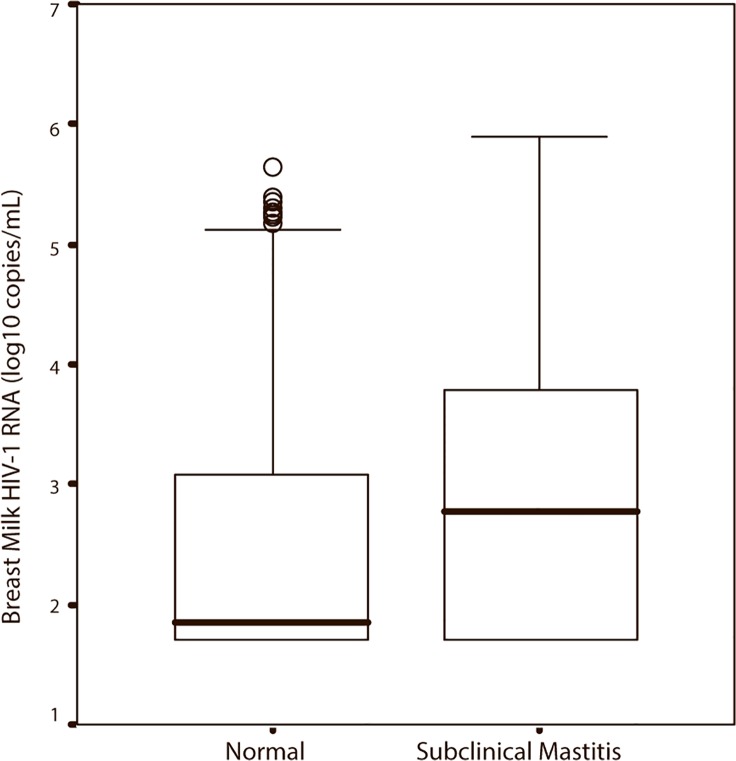
Breast Milk HIV-1 RNA in Samples from Breasts with and without Subclinical Mastitis. Subclinical mastitis is defined as sodium (Na^+^) concentrations >12 mmol/L. All breast milk samples with HIV-1 RNA and Na^+^ concentrations were used (744 samples with Na^+^ concentration ≤ 12 mmol/L and 453 samples with Na^+^ concentration >12 mmol/L). Box plots display medians, quartiles and outliers.

Of the 273 women participating in this study, breast problems were reported by 31/244 (13%) of the women during 6 weeks and most problems were reported at 1 week postpartum. Subclinical mastitis, defined in our study as Na^+^ concentrations >12 mmol/L, was detected in 182/273 (67%) of the women at some time during 6 weeks postpartum, and in 453/1,197 (38%) of the samples analyzed for Na^+^ concentration. The proportion of women with subclinical mastitis declined sharply over the 6-week period: from 74% (152/206) at 1 week, to 49% (98/198) at 4 weeks and to 34% (71/208) at 6 weeks postpartum. Overall, the majority of the women only had unilateral subclinical mastitis (48% at 1 week, 68% at 4 weeks and 85% at 6 weeks). Also, the median Na^+^ concentration in breast milk was significantly higher at 1 week compared to 4 weeks and 6 weeks postpartum (all P < 0.05) (Table 3). Breast milk samples with subclinical mastitis had significantly higher HIV-1 RNA at 1 week, 4 weeks and 6 weeks postpartum (all P < 0.05). When analyzing the breast milk samples from women with subclinical mastitis at 1 week, the sample from the contralateral breast did not have significantly higher HIV-1 RNA (P > 0.05). However, at 4 weeks postpartum HIV-1 RNA was significantly higher also in the contralateral breast (P = 0.02) and at 6 weeks postpartum there was a tendency towards higher HIV-1 RNA in the contralateral breast (P = 0.05). Breast milk HIV-1 RNA was also compared to Na^+^ concentration expressed as a continuous variable and HIV-1 RNA and Na^+^ concentration correlated at 1 week (R = 0.30, R = 0.29; all P = 0.01), 4 weeks (R = 0.42, R = 0.39; all P = 0.01) and 6 weeks postpartum (R = 0.37, R = 0.35; all P = 0.01) The association between Na^+^ concentration and breast milk HIV-1 RNA is shown in Fig. 4. Plasma HIV-1 RNA in the group with subclinical mastitis and the group with normal Na^+^ concentration in the breast milk was also compared, but no significant difference in plasma HIV-1 RNA was found at any time between the groups.

**Table 3:** Sodium Concentration in Bilateral Breast Milk Samples from 273 HIV-Infected Women.

	Breast Milk Right (mmol/L)	Breast Milk Left (mmol/L)
1 Week	n = 201	n = 200
Median, (range)	15 (5–107)	13 (5–110)
4 Weeks	n = 196	n = 193
Median, (range)	10 (5–112)	10 (5–106)
6 Weeks	n = 202	n = 205
Median, (range)	5 (5–107)	5 (5–107)

## Discussion

In this study we examined factors affecting breast milk viral load in HIV-infected breastfeeding women, i.e. maternal plasma HIV-1 RNA, NVP and subclinical mastitis. Plasma and breast milk HIV-1 RNA dropped significantly after administration of short-course ARV prophylaxis. Moreover, subclinical mastitis had a marked effect on the viral load in the breast milk

Our findings are in line with a study that demonstrated high NVP concentrations through week 1 after single-dose NVP intrapartum, accompanied by suppression of plasma and breast milk viral load [21]. Moreover, we found detectable NVP concentrations in plasma in the majority of the women and infants at time of delivery. Additionally, traces of NVP were present in plasma for a minimum of 4 weeks postpartum, indicating that additional drugs are needed for longer than one week to avoid development of NNRTI resistance. We only found correlation between HIV-1 RNA and NVP in plasma at 1 week postpartum. This lack of correlation between NVP concentration and drop in viral load might be explained by the effect of the additional study drugs (i.e. Combivir twice daily for seven days or single-dose Truvada).

Our study confirmed the correlation between HIV-1 RNA loads in plasma and breast milk, with the lowest values found in breast milk [6;27;28]. We also confirmed the correlation of HIV-1 RNA loads between samples from the right and the left breast, respectively [27]. However, we found a significant difference in viral loads between breasts at 4 and 6 weeks postpartum. The concordance in viral load between breasts at 1 week postpartum is probably a consequence of the fact that the majority of the breast milk samples at this time had undetectable viral load due to the effect of ARV prophylaxis. Accordingly, the viral load in breast milk in the absence of ARVs seems to be more influenced by local factors, e.g. mastitis.

This study corroborated the impact of subclinical mastitis, reflecting epithelial permeability, on breast milk viral load [5;12;29]. Furthermore, and in keeping with others, we found that subclinical mastitis occurred most often early in the postpartum period [11]. However, elevated Na^+^ concentrations are physiological in the very early breast milk (and again at the termination of weaning) [30] and Semrau and colleagues demonstrated that elevated Na^+^ concentrations at 1 week postpartum were common and not associated with HIV transmission. By contrast, the study showed that elevated breast milk Na^+^ concentrations at four months were associated with HIV transmission [27]. Also, Filteau et al found a high portion of women with subclinical mastitis early in the postpartum period and furthermore demonstrated that the concentration of immune factors correlated significantly with the indicator of subclinical mastitis also at this point of time [31]. In our study, laboratory-based diagnosis of mastitis was supplemented with reported symptoms of breast problems and in concordance with the laboratory established mastitis, clinical breast problems also occurred early in the postpartum period. In addition, we established that subclinical mastitis is mostly unilateral, emphasizing that studies investigating the impact of mastitis with respect to MTCT should test breast milk from both breasts. The impact of Na^+^ concentration on HIV-1 RNA in breast milk was confirmed in this study. Interestingly we found a tendency towards higher HIV-1 RNA also in the contralateral breast. In contrast, a study investigating clinical mastitis did not observe such a change in the contralateral breast [11]. This is probably reflecting some lack of specificity in the laboratory-based diagnosis of mastitis.

Some limitations should be considered in interpreting our findings. Firstly, the main study was designed to assess the development of NVP resistance after two short-course ARV prophylaxis regimens designed to reduce the risk of NVP resistance. In the current study we did not take these different short-course regimens into account during the statistical analyses, however we tested that there were no differences in NVP, HIV-1 RNA or Na^+^ concentrations between the two trial arms in the ComTru study at any point of time. Secondly, the main study had a considerable loss-to-follow-up and this could potentially skew our results. We decided to include all women who provided minimum one breast milk sample in the current analysis. The strengths of the study include the very large number of breast milk samples analyzed. Moreover, the study is strengthened by the advantage that breast milk samples were taken from both breasts in most instances, whereas others have tested only unilaterally. Our results show that bilateral sampling is crucial to investigate the impact of mastitis on viral load in breast milk. In addition, we used a novel, low-cost and easy-to-use assay for NVP-testing, allowing us to measure all breast milk samples with expected detectable concentrations, and not just a subset as have been done by others. Conversely, the assay used has a detection limit that is markedly higher than the more costly HPLC method. However, the main purpose of these measurements was to establish whether NVP was detectable (and thereby above the value considered to be protective to MTCT) and not the concrete value. Because we measured NVP in samples from both breasts we might have a higher rate of detectable samples than other studies.

Our analysis should be viewed in the context of the former guidelines on PMTCT based on single-dose NVP. In recent years impressive progress has been made in scaling-up the use of ART for treating and preventing HIV infection [19]. The single-dose NVP regimens are now less used but NVP continues to be a backbone in ARV prophylaxis where triple ARVs are not feasible. Even though this option is last resort, NVP-based regimens might have eligibility in places where triple ARV prophylaxis is simply not an alternative.

In conclusion, this study confirms that there are many factors affecting the viral load in breast milk, including plasma viral load, NVP and subclinical mastitis. NVP was detectable in nearly all infant cord blood samples after single-dose NVP and the NVP concentration in maternal plasma and breast milk remained high through week 1 postpartum accompanied by suppressed HIV-1 RNA in plasma and breast milk. Furthermore, breast milk samples with subclinical mastitis had significantly higher breast milk HIV-1 RNA.

## Supporting Information

S1 Dataset (XLS)
